# Primary care physician referral patterns in Ontario, Canada: a descriptive analysis of self-reported referral data

**DOI:** 10.1186/s12875-017-0654-9

**Published:** 2017-08-22

**Authors:** Clare Liddy, Sadaf Arbab-Tafti, Isabella Moroz, Erin Keely

**Affiliations:** 1 C.T. Lamont Primary Healthcare Research Centre, Bruyère Research Institute, 43 Bruyere St, Annex E, Room 106, Ottawa, ON K1N 5C8 Canada; 2 Department of Family Medicine, University of Ottawa, 75 Laurier Ave E, Ottawa, ON Canada; 3 Faculty of Medicine, University of Ottawa, 75 Laurier Ave E, Ottawa, ON Canada; 4 Department of Medicine, University of Ottawa, 75 Laurier Ave E, Ottawa, ON Canada; 5 Division of Endocrinology/Metabolism, The Ottawa Hospital, 1967 Riverside Drive, Ottawa, ON Canada

**Keywords:** Primary care, Referrals, Specialist care, Allied health

## Abstract

**Background:**

In many countries, the referral-consultation process faces a number of challenges from inefficiencies and rising demand, resulting in excessive wait times for many specialties. We collected referral data from a sample of family doctors across the province of Ontario, Canada as part of a larger program of research. The purpose of this study is to describe referral patterns from primary care to specialist and allied health services from the primary care perspective.

**Methods:**

We conducted a prospective study of patient referral data submitted by primary care providers (PCP) from 20 clinics across Ontario between June 2014 and January 2016. Monthly referral volumes expressed as a total number of referrals to all medical and allied health professionals per month. For each referral, we also collected data on the specialty type, reason for referral, and whether the referral was for a procedure.

**Results:**

PCPs submitted a median of 26 referrals per month (interquartile range 11.5 to 31.8). Of 9509 referrals eligible for analysis, 97.8% were directed to medical professionals and 2.2% to allied health professionals. 55% of medical referrals were directed to non-surgical specialties and 44.8% to surgical specialties. Medical referrals were for procedures in 30.8% of cases and non-procedural in 40.9%. Gastroenterology received the largest share (11.2%) of medical referrals, of which 62.3% were for colonoscopies. Psychology received the largest share (28.3%) of referrals to allied health professionals.

**Conclusion:**

We described patterns of patient referral from primary care to specialist and allied health services for 30 PCPs in 20 clinics across Ontario. Gastroenterology received the largest share of referrals, nearly two-thirds of which were for colonoscopies. Future studies should explore the use of virtual care to help manage non-procedural referrals and examine the impact that procedural referrals have on wait times for gastroenterology.

**Electronic supplementary material:**

The online version of this article (doi:10.1186/s12875-017-0654-9) contains supplementary material, which is available to authorized users.

## Background

In many countries, referrals from primary care providers (PCPs) to specialists are a necessary step for patients to access health resources. However, the referral process faces a number of challenges from inefficiencies and rising demand, resulting in excessive wait times for many specialties [1, 2]. Referral patterns from primary care to specialty care have been previously studied in several countries—including Canada, the United States, and the United Kingdom—using a range of data sources, including chart audits, surveys, health administrative databases, and electronic health records [3–8]. However, differences in local contexts, study methods, and measures of referral patterns make it difficult to compare results between studies. Variations in health system structure make comparisons a particular challenge, as patients in the United States can access secondary care directly while patients in countries with universal healthcare must often access such services through referral by their PCP [4, 7, 8]. While a few studies have examined referral patterns in Canada, their findings are several years old and drawn from health administrative databases, which cannot paint a complete picture of referral activities at a given clinic [3–5, 9, 10].

Therefore, as part of a larger program of research examining referral issues, wait times, and the use of electronic consultation (eConsult) to improve access to specialist advice, we collected referral data from a sample of family doctors across the province of Ontario [11]. This study describes referral patterns to specialty services using PCP self-reported patient referral data. To our knowledge, this is the first study to explore referral patterns using this type of raw, practice-derived data, which allows for a unique study of referrals made not only to medical specialists but also to allied health professionals. PCP referral patterns may be of interest to healthcare providers, health system administrators, and policy makers, as they reflect the ever-changing supply and demand for various services and are significant drivers of healthcare costs. Knowledge of these patterns can help inform health care funding decisions and resource allocation.

## Methods

### Design

We conducted a prospective study of referral patterns from PCPs to specialist and allied health services using self-reported de-identified patient referral data from participating PCPs across Ontario collected over a 20-month period (June 2014–January 2016).

### Population

PCPs were recruited as part of a larger cluster randomized controlled trial evaluating the impact of the Champlain BASE™ (Building Access to Specialists through eConsultation) service—a novel electronic referral-consultation process—on overall specialist referral rates. All PCPs practicing in Ontario who were not already enrolled with eConsult were eligible to participate in the study. Details of the recruitment process have been published elsewhere [11]. Participating PCPs were invited to submit monthly patient referral data on a voluntary basis as part of the trial.

### Setting

All PCPs came from Ontario. The province has a population of 13 million people with health outcomes and demographic characteristics comparable to the rest of Canada [12].

### Data collection

Data was prospectively collected using a standardized referral tracking form (Additional file 1) adapted from a similar tool obtained from the American Academy of Family Physicians [13]. The form included month of referral request, type of specialty, reason for referral, and whether the referral was for a procedure. The referral tracking data were faxed or emailed to the research team on a monthly basis and entered into a database by a research assistant.

Information on PCP demographics (gender, year of graduation, and medical education location) was obtained from the College of Physicians and Surgeons of Ontario (CPSO) website. Clinics completed a survey adapted from two validated Pan-Canadian Primary Health Care Provider and Practice Surveys from the Canadian Institutes for Health Information, [14] which inquired about demographic characteristics (postal code, primary setting, years in operation, number of PCPs and presence of on-site specialist services), Electronic Medical Record (EMR) use, referral method, and presence of a designated staff for scheduling/tracking referrals or liaising with specialist offices. The Rurality Index for Ontario (RIO) 2008 score was calculated using the Ontario Medical Association (OMA) RIO postal code look-up and used to categorize clinics into rural (score = 0–10), semi-urban (score = 10–40), and urban (score = 40–100) settings.

### Data analysis

All PCPs who submitted at least 6 months of referral data were included in the analysis. Referrals that did not occur face-to-face (e.g. eConsults) or did not indicate a target specialty were excluded.

Descriptive statistics were generated to identify the most frequently accessed services, the reason for referral, and whether referrals were procedural. As referral volumes per month did not follow a normal distribution, the number of referrals per PCP per month was reported using medians and interquartile ranges.

## Results

**Fig. 1:**
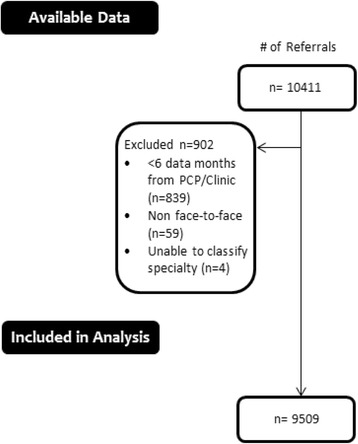
Referral data

A total of 9509 referrals submitted by 30 PCPs from 20 clinics were eligible for analysis (Fig. 1 **)**.

### Respondent characteristics

Table 1 provides descriptive characteristics of PCPs and clinics. Most PCPs were female (63%) and trained in Canada (90%). Most clinics were established urban (70%) group practices (76%) without access to on-site specialist services (71%). Of the five clinics with access to on-site specialist services, three clinics had only one medical specialist while the other two clinics had two and eight medical specialists, respectively. Specialty type was not specified. All clinics reported using EMRs to order tests and prescribe medication, and 77% indicated they used them to make referrals. However, 40% of clinics reported completing referrals using a combination of paper-based and electronic methods, and 17% referred by paper alone.

**Table 1:** Respondent characteristics

PCP (*N* = 30) Characteristics	
Gender	
Female*, n (%)*	19 (63.3)
Male*, n (%)*	11 (36.7)
Medical Training University Location	
Canada*, n (%)*	27 (90)
Outside Canada*, n (%)*	3 (10)
Years Since Graduation	
*Mean (SD)*	24.8 (11.3)
# of Referrals per PCP per Month	
*Median (IQR)*	26 (11.5–31.8)
Clinic (*N* = 20) Characteristics	
Location (*N* = 20)	
Rural*, n (%)*	1 (5)
Semi-Urban*, n (%)*	5 (25)
Urban*, n (%)*	14 (70)
Primary Setting (*N* = 17)	
Primary Solo Practice*, n (%)*	0
Physician Group Practice*, n (%)*	13 (76.5)
Community Clinic/Community Health Centre*, n (%)*	1 (5.9)
Walk-In Care Centre/Clinic*, n (%)*	0
Clinic Affiliated with Hospital/Ambulatory Care Unit*, n (%)*	0
University Clinic or Teaching Unit*, n (%)*	3 (17.6)
Other*, n (%)*	0
Years in Operation (*N* = 17)	
Less than 1 Year*, n (%)*	0
1 to 4 Years*, n (%)*	2 (11.8)
5 to 9 Years*, n (%)*	3 (17.6)
More than 10 Years*, n (%)*	12 (70.6)
# of Primary Care Providers per Clinic (*N* = 17)	
*Mean (SD)*	6.4 (6.0)
On-Site Specialists Services (*N* = 17)	
Yes*, n (%)*	5 (29.4)
No*, n (%)*	12 (70.6)
EMR Use (*N* = 17)	
Yes	17 (100)
Electronic ordering of tests*, n (%)*	17 (100)
Electronic prescribing of medication*, n (%)*	17 (100)
Electronic referrals directly to specialists*, n (%)*	13 (76.5)
Viewing electronic reports of patients test results ordered by you or your practice*, n (%)*	16 (94.1)
Viewing electronic reports of patients test results ordered by another provider outside your practice*, n(%)*	15 (88.2)
Viewing electronic reports of patients hospital records*, n (%)*	15 (88.2)
Viewing electronic reports of patient imaging results*, n (%)*	14 (82.4)
Other*, n (%)*	1 (5.9)
No, but Planning to Adopt within a Year*, n (%)*	0
No*, n (%)*	0
Referral Method (*N* = 17)	
Electronic*, n (%)*	6 (35.3)
Paper*, n (%)*	3 (17.6)
Both*, n (%)*	7 (41.2)
Other*, n (%)*	1 (5.9)
Designated Staff for Scheduling Referrals (*N* = 17)	
Yes*, n (%)*	17 (100)
No*, n (%)*	0
Designated Staff for Liaising with Specialist Office to Follow-Up on Patient Visits (*N* = 17)	
Yes*, n (%)*	14 (82.4)
No*, n (%)*	3 (17.6)
Designated Staff for Tracking Referrals (*N* = 17)	
Yes*, n (%)*	16 (94.1)
No*, n (%)*	1 (5.9)

### Referral patterns

**Fig. 2:**
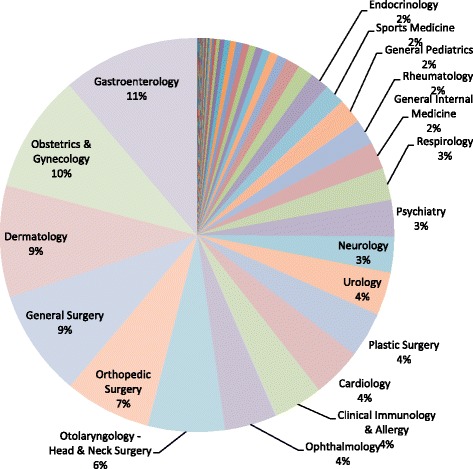
Medical specialty referral distribution (*N* = 9297)

PCPs completed a median of 26 (interquartile range 11.5 to 31.8) referrals per month. Ninety-eight percent of included referrals (*n* = 9297) were directed to medical professionals while only 2% (*n* = 212) were directed to allied health professionals. Distribution of all medical specialty referrals is shown in Fig. 2. Pediatric specialty referrals made up 2.8% (*n* = 261) of medical specialty referrals.

Of the 9297 referrals directed to medical professionals, 55% (*n* = 5118) were directed to non-surgical specialties while 45% (*n* = 4167) were directed to surgical specialties. The top non-surgical specialty referrals were identified as gastroenterology (20.4%, *n* = 1042), dermatology (16.7%, *n* = 854), clinical immunology & allergy (7.4%, *n* = 380), cardiology (7.4%, *n* = 378), neurology (5.4%, *n* = 277), psychiatry (5.4%, *n* = 276), respirology (4.8%, *n* = 218), general internal medicine (4.1%, *n* = 208), rheumatology (3.8%, *n* = 196) and general pediatrics (3.7%, *n* = 187).

Among referrals to medical specialists, 30.8% were identified as procedural, 40.9% as non-procedural, and 28.3% were unspecified (Table 2). More than half of all referrals to gastroenterology, obstetrics and gynecology, general surgery, and plastic surgery were identified as procedural. Colonoscopy made up 62.3% of all gastroenterology referrals and 24.1% of general surgery referrals.

**Table 2:** Top 10 medical specialty referrals by procedure (*N* = 9297)

	Total	Gastro-enterology	Plastic surgery	General surgery	Obstetrics & gynecology	Clinical immunology & allergy	Orthopedic surgery	Dermatology	Otolaryngology (Head & neck surgery)	Cardiology	Ophthal-mology
Yes	2927 (30.8)	764 (73.3)	205 (59.2)	432 (53.1)	460 (50.9)	143 (37.6)	223 (33.8)	157 (18.4)	101 (17.2)	25 (6.6)	25 (6.3)
No	3889 (40.9)	206 (19.8)	44 (12.7)	46 (5.7)	308 (34.1)	176 (46.3)	124 (18.8)	572 (67)	336 (57.3)	208 (55)	73 (18.5)
NS	2693 (28.3)	72 (6.9)	97 (28)	336 (41.3)	135 (15)	61 (16.1)	312 (47.3)	125 (14.6)	149 (25.4)	145 (38.4)	296 (75.1)

*NS* Not specified

**Fig. 3:**
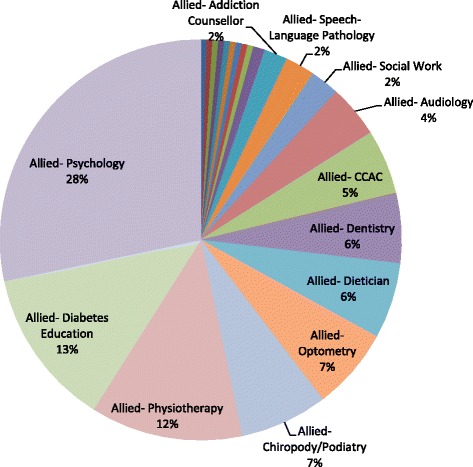
Allied health referral distribution (*N* = 212)

Distribution of allied health referrals is shown in Fig. 3. The top five were identified as psychology, diabetes education, physiotherapy, chiropody/podiatry, and optometry.

## Discussion

The most frequently referred-to specialty was gastroenterology, followed closely by obstetrics and gynecology, dermatology, and general surgery. Of the specialties that received mostly procedural referrals, gastroenterology was again identified as the top specialty, with colonoscopy accounting for nearly two-thirds of gastroenterology referrals and a quarter of general surgery referrals. Not surprisingly, gastroenterology and dermatology are also among the specialties with the longest wait times in Ontario [9, 10]. This information should be used to inform and plan solutions to improve wait times.

To our knowledge, this is the first descriptive study of referral patterns in Canada using PCP-derived, self-reported patient referral data. Other studies of referral patterns have focused mostly on examining referral rates and on trying to understand the key factors affecting them. Their findings demonstrate substantial variability in referral rates related to physician, patient, practice, community, and healthcare system characteristics, and present a general lack of consensus regarding which type of factors account for the most of the observed variability in referral rates [3–5, 15–19]. Very few of these studies reported on the distribution of referrals coming from primary care, though many aligned with our findings in terms of which specialties received a preponderance of referrals [3, 9, 18, 19]. One study also reported gastroenterology as the most frequently referred-to specialty, [19] while others cited dermatology [9, 18] and general surgery [3]. These findings suggest recurring patterns, though caution must be taken when comparing studies due to variations in setting and methodology.

Studies based in other countries have detected similar referral patterns [20–22]. An Australian study of general practices examined which specialty groups received the most referrals. Their findings mirrored ours in many respects, with several of their top ten specialties—notably orthopedic surgery, general surgery, gastroenterology, and dermatology—appearing among ours as well, albeit in a different order [20]. Their reported patterns of allied health referral were likewise similar, citing physiotherapy, psychology, diabetes education, chiropody/podiatry, and optometry [20]. When exploring reasons for referral to gastroenterology, the most commonly cited were rectal bleeding and digestive neoplasm [20]. This suggests that a high proportion of referrals to gastroenterology in Australia may also be for colonoscopy as these presentations lend themselves to further investigation. Another general practice study out of England discussed the impact of prevention-based programs on overall wait times, suggesting that “new published guidelines on suspected cancer recognition and referral lowered referral thresholds requiring general practitioners to refer many more people with non-specific or early signs of possible cancer” [22].

The fact that procedural referrals such as colonoscopies represented such a high proportion of referrals in our study—particularly to gastroenterology—raises concerns about the impact of prevention-based programs on overall wait times. While well-intentioned, these programs may be generating an overly large volume of referrals for such procedures and thus may require specific strategies to enable timely access to colonoscopy for patients with a time-sensitive diagnosis, such as colorectal cancer. A nationwide practice audit of wait time for gastroenterology care revealed a median wait time (from referral to procedure) of 91/203 days (median/75th percentile) for Canada and 72/118 days for Ontario [3]. Furthermore, median wait times were 99/208 days for physicians who offered screening colonoscopy for average-risk patients versus 66/180 days for physicians who did not [3]. These wait times greatly exceed the 2006 benchmarks set by the Canadian Association of Gastroenterology [23] and may come with substantial costs, as digestive diseases account for 15% of the health care spending in the Ontario, exceeding all other disease categories [24]. While referrals to general surgery have minimized some of the procedural burden, the number of colonoscopy referrals to gastroenterology remains high, and requires a targeted and more efficient management strategy.

Non-procedural referrals may lend themselves to virtual care in some settings. Forty percent of referrals in our study were non-procedural and thus may have been eligible to be handled via telemedicine or eConsult services. These services have the potential to address excessive wait times for specialist care, which are a serious issue in Canada; a recent survey by the Commonwealth Fund placed Canada last in timeliness of care among the 11 countries surveyed [25]. Prolonged waiting for specialist care can cause patients anxiety, delay important diagnoses and treatments, and lead to poorer health outcomes [2, 26]. eConsult services have demonstrated effectiveness at improving access, increasing patient and provider satisfaction, and lowering costs [27, 28]. However, such services are not self-implementing and require deliberate uptake by clinics and providers. Potential challenges in this regard are reflected in participating clinics’ incomplete adoption of EMR-based referral systems with over one-third reporting the use of paper and electronic means to refer. The hesitance to switch to exclusively electronic referral methods stems from many factors, including provider preferences and the fact that EMRs from different vendors are unable to communicate with each other [29].

We also found that a small but sizeable number of referrals were made to allied health services, of which psychology was the most frequent. Unlike with medical specialty services, patients can access allied health services without first being referred by a PCP. At present, only one-third of patients in Ontario have access to publically-funded allied health services [30]. Patients outside of this group must rely on private insurance to cover costs or else pay for services out of pocket, putting lower income patients at risk of experiencing poorer access to care. Further work is needed to explore potential inequities in access to allied health services and whether or not they have an impact on patient health outcomes.

Our study has several limitations. Our data collection strategy did not allow us to report referral rates or examine patient, provider, and clinic factors related to the observed referral patterns. Participation was voluntary and consisted of a convenience sample of PCPs interested in gaining access to the eConsult service, hence introducing a selection and possibly a response bias. Most participating clinics were in the central, eastern and western regions of the province and all had access to an EMR. This in turns limits generalizability of the results, specifically for more rural practices in northern Ontario. There was also no mechanism to verify whether the participating PCPs reported all referrals, especially to the allied health providers. As such the number of allied health referrals may actually be an underestimate.

## Conclusion

We examined patterns of patient referral from primary care to specialist and allied health services for 30 PCPs in 20 clinics across Ontario. Future studies should explore the use of eConsults and other forms of virtual care to help manage non-procedural referrals and examine the impact that procedural referrals have on wait times for gastroenterology. A better understanding of when and why PCPs referral to allied health professionals—particularly psychologists—is needed to ensure that patients receive access to essential care regardless of their level of income.

## Additional file

Additional file 1: Standardized tracking form used to collect referral data. We have provided a copy of the standardized tracking form that clinics used to collect and send data regarding patient referrals. (DOCX 45 kb)

## Abbreviations

BASE: Building access to specialists through eConsultation
PCP: Primary care provider
