# Supplementary figures and images for: Primary care physician referral patterns in Ontario, Canada: a descriptive analysis of self-reported referral data
Source: BMC Fam Pract. 2017 Aug 22;18:81. doi: 10.1186/s12875-017-0654-9 (PMC5567435; doi:10.1186/s12875-017-0654-9)

**Additional file 1** Standardized tracking form used to collect referral data

**
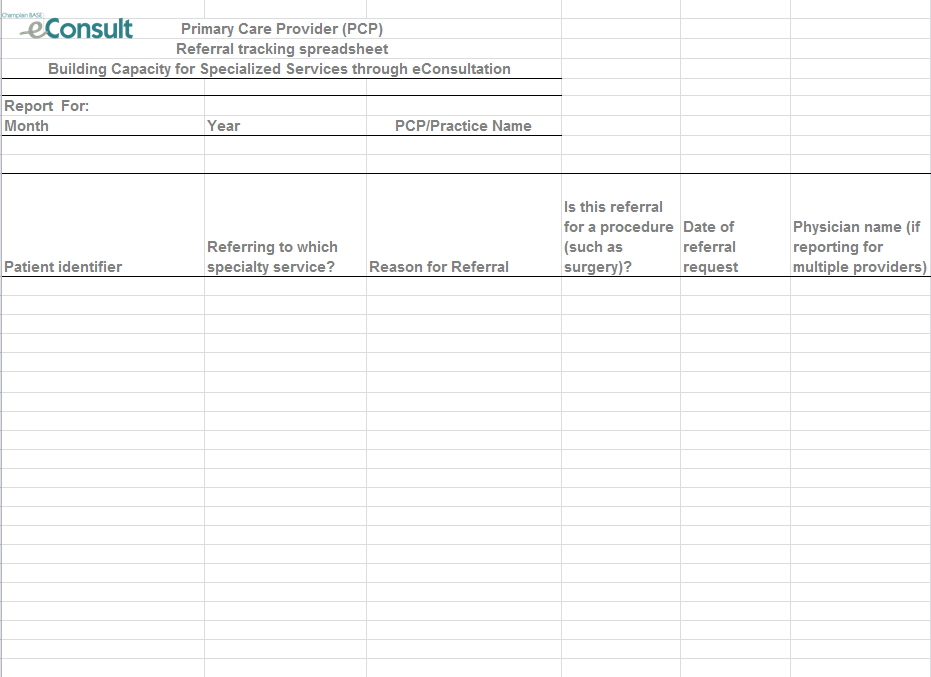
**

Supplement: Additional file 1: — Standardized tracking form used to collect referral data. We have provided a copy of the standardized tracking form that clinics used to collect and send data regarding patient referrals. (DOCX 45 kb) [file 12875_2017_654_MOESM1_ESM.docx]
